# Supplementary material for: Recommendations for clinical interpretation of variants found in non-coding regions of the genome
Source: Genome Med. 2022 Jul 19;14:73. doi: 10.1186/s13073-022-01073-3 (PMC9295495; doi:10.1186/s13073-022-01073-3)
Supplement: Supplementary file 2 — Additional file 2: Fig S1. Identifying regulatory variants in trans with pLoF variants in GEL. [file 13073_2022_1073_MOESM2_ESM.pdf]

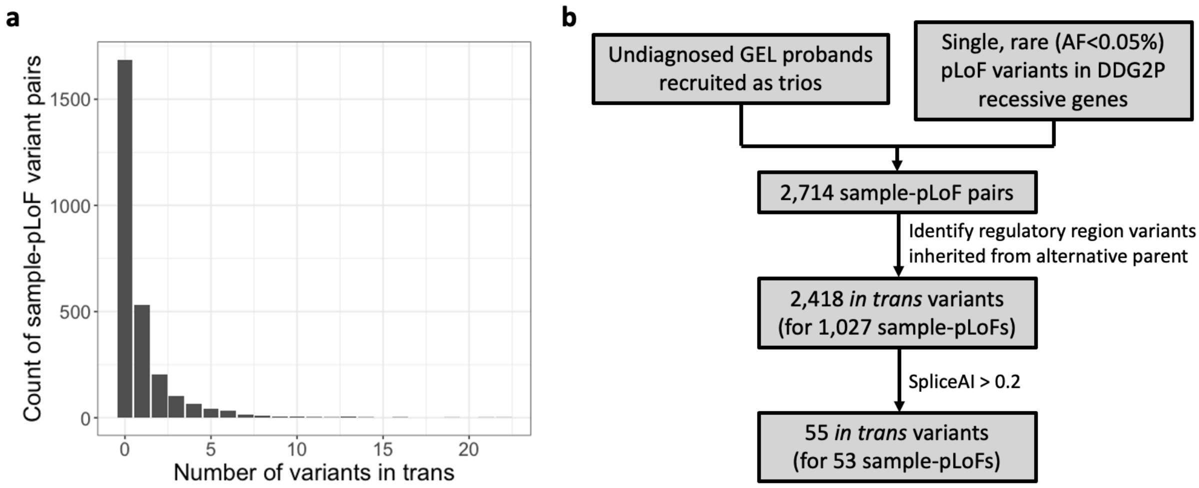

Fig S1: Identifying regulatory variants *in trans* with pLoF variants in GEL. (a) The number of variants identified in trans per sample-pLoF pair. One or more *in trans* variants were found for 37.8% of pLoF variants. (b) A flow diagram of the approach.
